# Obstacle Crossing Path Planning for a Wheel-Legged Robot Using an Improved A* Algorithm

**DOI:** 10.3390/s25185795

**Published:** 2025-09-17

**Authors:** Jinliang Lu, Ming Pi, Guoxin Zeng

**Affiliations:** School of Information and Control Engineering, Southwest University of Science and Technology, Mianyang 621010, China; kimliang@mails.swust.edu.cn (J.L.); zgx@mails.swust.edu.cn (G.Z.)

**Keywords:** wheel-legged robot, obstacle negotiation, A* algorithm, jump point, continuous jumping constraint mechanism

## Abstract

In response to the challenges of obstacle avoidance and terrain negotiation encountered by wheel-legged robots in static environments with complex obstacles, this study introduces an enhanced A* path planning algorithm that incorporates a jump-point search strategy, a dynamically weighted heuristic strategy, and a continuous jumping constraint mechanism to facilitate efficient obstacle traversal. The algorithm extends the traditional 8-neighborhood rule to support jumping in the horizontal, vertical, and diagonal directions. A dynamic, weighted heuristic is introduced to adaptively adjust heuristic weights, guide the search direction, improve efficiency, and reduce detours. Redundant point removal and Bézier curve smoothing were employed to enhance path smoothness, whereas the continuous jumping constraint limited the jump frequency and improved motion stability. The results validate that—relative to the standard A* algorithm, which achieves a 73.7% reduction in path nodes (from 54 to 16)—85% fewer search nodes (from 542 to 78) and a planning time of 0.0032 s were achieved while also enhancing performance in crossing complex structures. This enhances the capability of wheel-legged robots to perform real-time path planning in structurally complex yet static environments, thereby improving their autonomous navigation efficiency.

## 1. Introduction

Driven by the ongoing progress in robotics, wheel-legged robots, i.e., robots that harness the hybrid locomotion benefits of wheeled and legged systems [1], have attracted widespread attention due to their superior obstacle crossing ability [2] and adaptability in complex terrain. Wheel-legged robots integrate the high efficiency of wheeled locomotion with the terrain adaptability and flexibility of legged movement, and they perform particularly well in obstacle crossing tasks. However, in practical applications, implementing a stable and computationally efficient path planner in a dynamic environment [3] remains a challenge.

Path planning is typically achieved using methods such as graph traversal, sampling-based searches, and potential field algorithms. The A* algorithm [4] and the Djikstra algorithm [5] are typical graph search algorithms, and the artificial potential field method [6] is a typical implementation of potential field strategies. Random tree [7] is a typical sampling search algorithm that has been broadly adopted for pathfinding in static environments. For example, Wu et al. [8] combined an improved random tree algorithm with the dynamic window method and combined the idea of an artificial potential field to reduce redundant points, thereby significantly improving the efficiency of Unmanned Aerial Vehicles (UAVs) to avoid static and dynamic obstacles. Reinforcement Learning (RL), as a model-free method, has unique advantages in path planning. Through the agent’s autonomous learning of the optimal strategy, it performs outstandingly in scenarios such as dynamic obstacle avoidance and multi action collaborative planning. Typical algorithms include Q-learning [9], DDPG [10], and others. For example, Castañeda et al. [11] used an actor–critic approach to overcome the path planning problem of mobile robots that fully cover the environment. Similarly, addressing navigation in complex environments, Zhang et al. [12] proposed a hierarchical path planning method for wheeled mobile robots in partially known uneven terrain using the A* algorithm for global pathfinding and Q-learning for local obstacle avoidance.

The conventional A* algorithm is characterized by inherent limitations, such as low efficiency, large search range, and poor avoidance of dynamic obstacles [13]. This can impede performance in complex environments. Many scholars have improved this concept. Yao et al. [14] enhanced the performance of the A* algorithm by integrating bidirectional search, the D* Lite algorithm, dynamic obstacle trajectory prediction, and the bat clustering algorithm. The experimental results demonstrated that the proposed approach outperformed traditional algorithms in terms of path planning performance. Qi et al. [15] employed polynomial fitting to improve the smoothness of A* path generation, thereby enhancing the motion comfort of firefighting robots. Ge et al. [16] designed a dual-estimation heuristic function based on both energy consumption and distance, and experiments demonstrated that their method achieved advantages in reducing both the trajectory length and power consumption. Liu et al. [17] adopted phase-specific heuristic functions combined with the artificial potential field method, and comparisons with multiple traditional path planning methods showed significant improvements in path length, number of searched nodes, and time efficiency. Meng et al. [18] used a cost evaluation function to improve search efficiency and combined it with model predictive control to achieve more efficient autonomous parking. Xu et al. [19] added unknown path costs to upgrade A* algorithm to strengthen its path planning capability in challenging and dynamic scenarios. Priya et al. [20] proposed a dynamic weighted A* algorithm to achieve stable operation of autonomous driving vehicles in dynamic environments in response to the problems faced by vehicles in dynamic environments. Fu et al. [21] used an improved A* algorithm with a new cost function to design global navigation routes for unmanned surface vessels operating in island and reef-dense regions, thereby improving navigation efficiency in island and reef areas. Xu et al. [22] targeted the path planning problem in a greenhouse by broadening the search neighborhood, employing the Floyd algorithm to optimize the path and improving accuracy and search speed. Saeed et al. [23] blended PID control logic into an A-based planning approach and adjusted the heading based on feedback, thereby enabling a ship to faster reach the optimal route underwater. Yu et al. [24] applied a refined A* algorithm incorporating adaptive extended convolution for UAV path planning, significantly decreasing both the number of explored nodes and the computation time. Despite its widespread use, the conventional A* algorithm is characterized by inherent limitations, such as low search efficiency and large search range, which can impede its performance in complex environments. Numerous scholars have proposed improvements that often focus on enhancing the search speed using bidirectional [14] or dynamic search methods [14,20], optimizing path smoothness using polynomial fitting [15], or reducing operational costs such as energy consumption [16]. Other advanced techniques, such as the receding horizon motion planning method proposed by Zhang et al. [25] for quadrotors, utilize B-splines and nonlinear optimization to generate safe and efficient trajectories for dynamic obstacle avoidance. Other studies have improved heuristic [16,17] or cost functions [18,19,21] to guide the search more efficiently, achieving better performance in terms of path length and computation time. A recent study by Sun et al. [26] further illustrated the advanced avoidance strategies for wheel-legged robots. They integrated Theta* and TEB algorithms, classifying obstacles to enable separate path planning for the robot’s body and wheels. Although this method effectively generates smoother and safer avoidance trajectories, it is fundamentally designed to navigate obstacles. The core logic does not intrinsically evaluate or incorporate high-cost, high-reward traversal actions, such as jumping, as a strategic alternative to circumnavigation. This highlights a critical distinction: our work focuses not only on maneuvering around obstacles, but also on equipping the planner with the decision-making capability to physically cross them when it proves more efficient. However, these general improvements primarily target obstacle avoidance [22,23,24] and do not equip a planner with the logic to handle the unique multi-modal capabilities of wheel-legged robots, specifically the decision to perform high-cost, high-reward actions, such as obstacle crossing [27].

This challenge is especially relevant for complex tasks such as obstacle negotiation. While some studies have explored multi-modal path planning, such as for flying cars [28] (which can switch between ground and air travel to overcome obstacles), these approaches are not directly transferrable to the specific kinematics and stability constraints of wheel-legged robots. Moreover, the core of obstacle crossing for legged systems involves not only a high-level path, but also low-level control for maneuver execution, such as determining foothold selection and developing robust crossing strategies for specific obstacles. De Luca et al. [29] developed a method for a hybrid-legged/wheeled robots to adaptively traverse various terrains. Their method uses primitive behaviors based on ground and obstacle geometries with a perception module that processes LiDAR data to identify terrain features. This enables the autonomous selection and sequencing of traversal behaviors. Chen et al. [30] presented a method to improve the locomotion efficiency of a quadrupedal robot, which is better at traversing terrain but less efficient than wheeled robots. This method uses an optimized stance and CNN-based foothold classifier. It was validated in stair climbing tests with the Pegasus robot. Our work focuses on the high-level path planning that must precede these actions, enabling the robot to strategically identify when and where a jump is more advantageous than a detour.

It is also important to distinguish our approach from the well-known Jump Point Search (JPS) algorithm, which is another significant optimization of A*. JPS accelerates pathfinding by identifying “jump points” that allow the search to bypass large areas of the grid, thereby pruning the search space. Harabor et al. [31] proposed a novel search strategy for pathfinding in uniform-cost grid environments. This algorithm identifies and expands specific nodes, called jump points, to achieve fast optimal path calculations with no memory overhead. This study demonstrates that using jump points can significantly accelerate the A* algorithm while always ensuring optimal solutions; however, these “jumps” are a conceptual search pruning mechanism and do not represent a robot’s physical movement. In contrast, our work introduces a physically grounded “jump” action corresponding to a wheel-legged robot’s actual obstacle crossing capability. Therefore, our use of “jump points” specifically refers to the start and end nodes of a physical traversal over an obstacle, a concept distinct from the search optimization nodes in the JPS framework.

Therefore, how to optimize the existing path planning algorithm to enhance its adaptability to the obstacle crossing demands of wheel-legged robots in complex terrain has become an important direction of current research. To address the issues of low efficiency, inability to perform obstacle crossing actions, and unsmooth, tortuous paths when traditional A* algorithms are used for path planning in wheel-legged robots, this paper proposes an improved A* path planning method specifically designed for wheel-legged robots. The motivation of this study is to enable robots to flexibly integrate obstacle avoidance and obstacle crossing behaviors, thereby enhancing their real-time adaptability and motion stability. The proposed method incorporates three core strategies: (i) an obstacle crossing mechanism based on 16-neighborhood expansion, supporting jumping actions in the horizontal, vertical, and diagonal directions; (ii) a dynamically weighted heuristic function combined with a continuous jumping constraint mechanism to balance search efficiency and path stability; and (iii) redundant point removal and Bézier curve smoothing to improve path continuity and smoothness.

The main contributions of this study are as follows:

(1) A jump-extended A* framework is designed to leverage the unique obstacle crossing capability of wheel-legged robots, enabling the efficient traversal of surmountable obstacles.
(2) A dynamic heuristic weighting mechanism and continuous jumping constraints were introduced to enhance search efficiency while ensuring stability.
(3) The proposed algorithm was validated through extensive simulations on complex grid maps, demonstrating significant improvements in path length, smoothness, and obstacle crossing capability compared with the traditional A* algorithm.

## 2. Materials and Methods

### 2.1. Environmental Model

Path planning for obstacle crossing and avoidance in wheel-legged robots was developed using a grid-based approach [32]. The grid environment model discretizes complex terrain and obstacle information into a regular two-dimensional grid. Each grid cell represents a discrete cell in the environment, which can effectively simplify the path planning problem.

**Figure 1:**
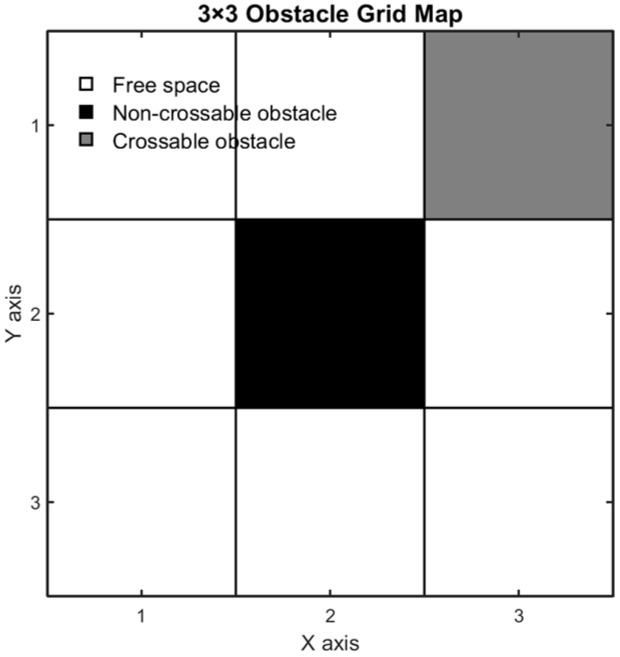
Simple obstacle map. Gray cells represent crossable obstacles, while black cells indicate impassable ones.

The model discretizes complex terrain into two-dimensional regular grids, each of which contain three types of states, as shown in Figure 1: free area (white), insurmountable obstacle (black), and surmountable obstacle (gray). Gray obstacles are permissible to cross, but the barrier-free conditions of the middle grid must be satisfied (e.g., diagonal crossings must be unobstructed in both adjacent directions). When planning the path, the improved A* algorithm integrates the 8-neighborhood obstacle crossing movement rules, giving priority to expanding the jump points across the gray obstacles while avoiding the black obstacles. The algorithm optimizes the search direction through a dynamic weighted heuristic function and marks the explored area, optimal path, and obstacle crossing points through a visual matrix.

### 2.2. Basic Theory of the A* Algorithm

In 1968, Hart et al. [33] proposed the A* algorithm by combining the Dijkstra and greedy search algorithms. It is frequently applied in path planning scenarios and graph traversal search algorithms. The value function is expressed as follows:

(1) f(n)=g(n)+h(n),

where *n* represents a specific node in the search space, *g*(*n*) denotes the actual cost of moving from the start node to node *n*, *h*(*n*) denotes the estimated heuristic cost from node *n* to the goal node, and *f*(*n*) denotes the comprehensive cost of node *n*.

Heuristic estimations are primarily derived from Manhattan, Euclidean, and diagonal distance calculations.

(2) $$h_{man}(n)=\left|x_{n}-x_{g}\right|+\left|y_{n}-y_{g}\right|,$$

(3) $$h_{eucl}(n)=\sqrt{\Delta x^{2}+\Delta y^{2}}\begin{array}{c} \;\;\;\;\;\;\;\;\Delta x=x_{n}-x_{g},\Delta y=y_{n}-y_{g} \end{array},$$

(4) $$h_{cheb}(n)={\left\|x_{n}-x_{g},\;y_{n}-y_{g}\right\|}_{\infty },$$

where (*x_n_*, *y_n_*) indicates the current position, and (*x_g_*, *y_g_*) indicates the goal position.

### 2.3. Obstacle Crossing Action Extension Mechanism

**Figure 2:**
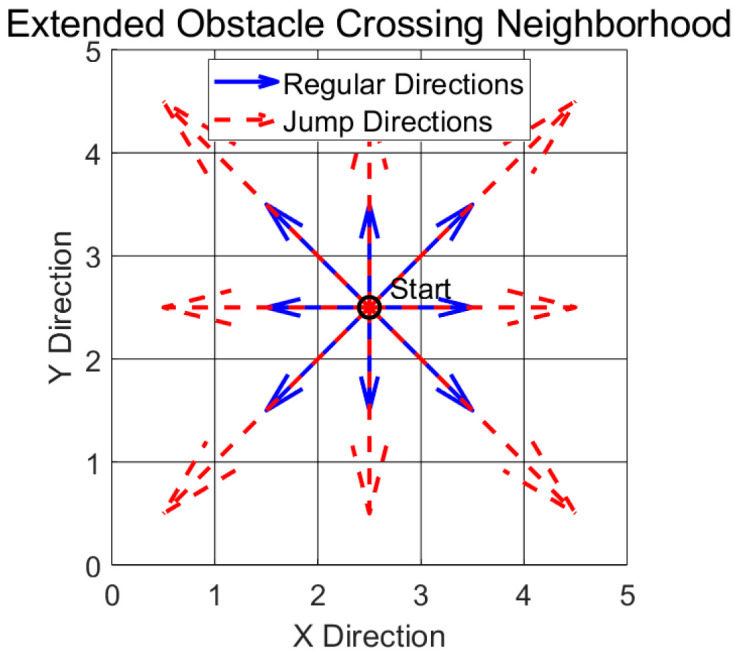
Extended obstacle crossing neighborhood. The blue solid arrows represent regular movement directions, while the red dashed arrows indicate jump directions. The central point denotes the current position (Start).

Jumping is a key obstacle crossing motion mode in the path planning of wheel-legged robots. The traditional A* algorithm extended to incorporate 8-neighborhood connectivity mainly serves the obstacle avoidance environment on the ground, and it is difficult to directly handle the interaction between the jumping trajectory and obstacles. To this end, this study adds an obstacle crossing mobility mechanism based on the original neighborhood and optimizes the safety constraints. In Figure 2, the neighborhood expansion is as follows:

The extended neighborhood includes eight standard moves and eight “jump” moves. A jump allows the robot to move over a single grid cell to land two cells away, horizontally, vertically, or diagonally. To ensure physical feasibility and to avoid collisions, every potential jump was evaluated against a strict set of rules: let the robot’s current node be at coordinates (*x*, *y*), then the validity of a jump depends on the status of the intermediate cell being jumped over and landing cell.

A horizontal jump from (*x*, *y*) to (*x* + 2, *y*) is possible if and only if the following apply: (1) The intermediate cell at (*x* + 1, *y*) must be a “crossable obstacle” (gray) and is an obstacle that needs to be surmounted. (2) The landing cell at (*x* + 2, *y*) must be “free space” (white). (3) The landing cell (*x* + 2, *y*) must be within map boundaries.

A diagonal jump from (*x*, *y*) to (*x* + 2, *y* + 2) has more complex safety requirements to prevent impassable obstacles. It is possible if and only if the following apply: (1) The intermediate cell at (*x* + 1, *y* + 1) must be a “crossable obstacle” (gray). (2) The landing cell at (*x* + 2, *y* + 2) must be “free space” (white). (3) The adjacent “corner” cells, (*x* + 1, *y*) and (*x*, *y* + 1), must not be “non-crossable obstacles” (black), which ensures that the robot has a clear path and does not collide with a wall or other impassable barriers during the jump. (4) The landing cell (*x* + 2, *y* + 2) must be within the map boundaries.

This detailed rule-based approach ensures that every potential jump is rigorously vetted for safety and feasibility before being considered by the path planner. The obstacle moving logic systematically applies these checks for all 8 possible jump directions from the current node.

**Figure 3:**
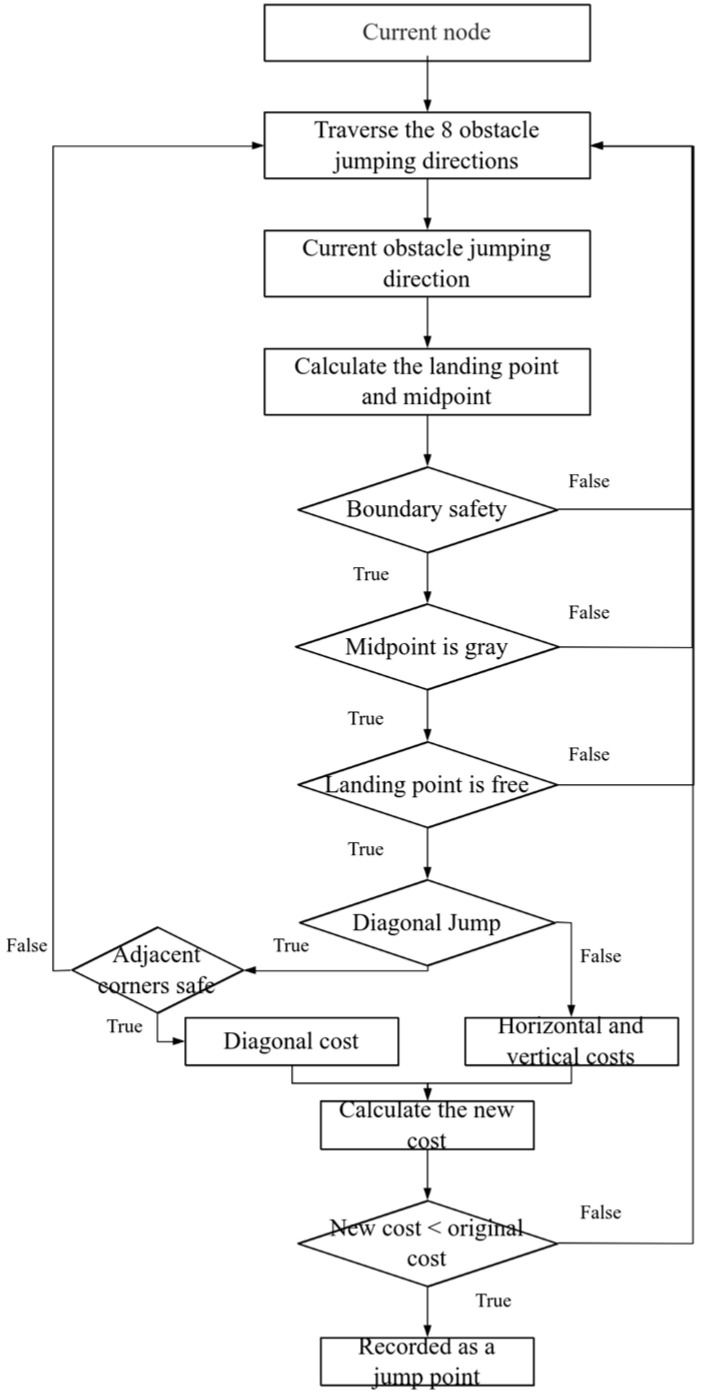
Obstacle overcoming movement logic flow diagram. The algorithm iteratively traverses eight possible jumping directions, checks boundary safety and obstacle properties, and computes costs for valid jumpable paths before expanding to the next node.

As presented in Figure 3, the logic for crossing obstacles is shown.

The obstacle moving logic is as follows.

Step 1: For the current node, iterate through the eight potential jump directions (horizontal, vertical, and diagonal) to determine the coordinates of the intermediate and landing nodes.

Step 2: Check the boundaries to ensure that all coordinates are within the map; otherwise, this jump direction is invalid.

Step 3: Check the attributes of the intermediate and landing nodes based on the rules for horizontal/vertical or diagonal jumps as described above. A jump is only valid if the intermediate node is a crossable (gray) obstacle and the landing node is free space (white). Otherwise, the node is abandoned, and Step 1 is executed again.

Step 4: For a diagonal jump, perform an additional collision avoidance check to ensure that the adjacent corner nodes are not non-crossable (black) obstacles.

Step 5: If all checks for a jump direction are passed, calculate the cost of this move. If this new path to the landing node is cheaper than any existing path, it is recorded as a potential jump point and the node’s cost is updated. Otherwise, return to Step 1.

Step 6: Process other directions and repeat Steps 1–5 until all directions are processed.

Jump points are exclusively recorded when the robot lands after crossing a gray obstacle. This strategy of expanding the neighborhood by integrating jumps enables the robot to both effectively avoid obstacles in complex environments and flexibly respond to obstacle crossing tasks, thus improving the robot’s adaptability in static environments with complex obstacles.

### 2.4. Dynamically Weighted Heuristic Function, Path Cost, and Admissibility Analysis

When facing intricate obstacle terrains, the traditional A* algorithm can be inefficient because of its fixed heuristic weight. To overcome this, a dynamic weighting strategy, which adaptively adjusts the influence of the heuristic function during the search, was adopted. In this strategy, the dynamic weight, ω(n)=1+α⋅(r/R), is defined and applied to the standard Euclidean heuristic, $h_{ecul}(n)$. The influence of the heuristic term is adjusted using this dynamic weight, where *α* is the dynamic adjustment coefficient used in the calculation. The resulting weighted heuristic, $h^{\prime }(n)$, is calculated using the following core formula.

When facing intricate obstacle terrains, the traditional A* algorithm has difficulty in achieving optimal search efficiency owing to fixed weights; therefore, a dynamic weighting strategy for the heuristic function is introduced, as shown in Equation (3). In this strategy, the influence of the heuristic term is adjusted using the dynamic weight (1+α·(r/R)), where *α* is the dynamic adjustment coefficient. The core formula is as follows:

(5) $$f(n)=g(n)+h^{\prime }(n),$$

(6) $$h^{\prime }(n)=(1+\alpha \cdot \frac{r}{R})\cdot h_{ecul}(n),$$

where *r* and *R* are the Euclidean distances from the present state to the target location and from the starting point to the target location, respectively; and *α* is the dynamic adjustment coefficient. At the beginning of the search, priority is given to finding the nearest path to increase search speed and reduce detours. When approaching the endpoint, search accuracy was improved. When gray obstacles that can be crossed are encountered, priority is given to the nodes that can be jumped over to reduce the path length.

It is important to analyze the impact of this dynamic weighting on the theoretical properties of the A* algorithm, specifically its admissibility. An algorithm is admissible if it is guaranteed that it will find the optimal path. Standard A* maintains admissibility when its heuristic function, *h*(*n*), never overestimates the true cost of the goal. In our method, the weight *ω* is greater than 1 when *α* > 0. This makes the heuristic $\omega (n)\cdot h_{ecul}(n)$ inadmissible as it may overestimate the true cost.

This modification transforms the algorithm into a weighted A*, which intentionally trades optimality for speed. For robotic applications requiring real-time path planning, finding a computationally efficient, near-optimal path is often more critical than guaranteeing the absolute shortest path at a higher computational cost. Parameter *α* allows us to control this trade-off, and a detailed sensitivity analysis to determine its value is presented in Section 3.5.

For the path cost term, the costs are assigned based on the type of node transition. For standard movements, the step cost is calculated using the Euclidean distance. For obstacle crossing (jump) movements, a higher fixed cost *c_jump_* was defined. This cost is intentionally set to be approximately twice that of the standard single-grid movement cost to reflect the increased energy expenditure and kinematic complexity associated with jumping actions. The act of jumping across a grid cell is kinematically equivalent to moving across two grid cells conventionally. Therefore, the cost for horizontal or vertical jumps is set to *c_jump_* = 2.0, which is twice the cost of a single horizontal/vertical movement. Similarly, diagonal jumps are equivalent to two consecutive diagonal movements, and their cost is set at *c_jump_* ≈ 2.8 to reflect the corresponding higher expense. A high cost makes the obstacle crossing mechanism useless as the robot will always prefer a long detour. Our chosen medium cost of approximately twice the standard movement cost provides a desirable balance, enabling the robot to use jumps strategically to significantly improve path efficiency while acknowledging that jumping is a more demanding action.

(7) $$g(n)=\left\{\begin{array}{l} g(p)+{\left\|n-p\right\|}_{2}\;\mathrm{if}\;\mathrm{standard}\;\mathrm{movement}\\ g(p)+c_{jump}\;\mathrm{if}\;\mathrm{jump}\;\mathrm{movement} \end{array}\right.,$$

where *g*(*p*) denotes the cumulative path cost of the parent node, and ${\left\|n-p\right\|}_{2}$ represents the Euclidean distance between node *n* and its parent node *p*.

### 2.5. Continuous Obstacle Crossing Constraint Mechanism

However, the dynamic weighting mentioned in the previous section may lead to problems with continuous jumping. In the early stages of the search, a larger weight *ω* makes the algorithm more “greedy,” favoring paths that advance rapidly toward the goal, which can include a series of consecutive jump actions. For a physical wheel-legged robot, continuous, high-frequency jumping significantly increases energy consumption and motion instability, potentially leading to mission failure. To strike a balance between the search efficiency and dynamic stability of a robot, it is crucial to introduce a mechanism that constrains this continuous jumping behavior.

**Figure 4:**
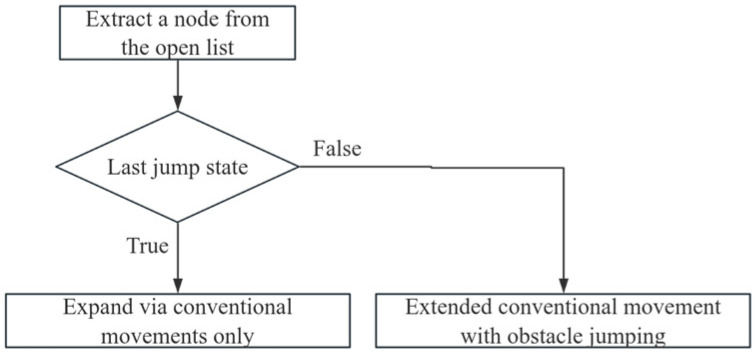
Flow diagram of the continuous obstacle protection mechanism.

To address the potential for the continuous jumping introduced by the dynamic weighting discussed in the previous section, a state-driven continuous obstacle crossing constraint mechanism was embedded in the improved A* algorithm to ensure the robot’s motion stability. When the robot reaches a new target point through obstacle crossing, the node is marked as the last jump. If it is false, the next step is to perform expansion in the conventional and obstacle crossing directions; if it is true, the next step is restricted to conventional movement only. This mechanism constrains the continuous obstacle crossing action. Figure 4 presents the flowchart.

### 2.6. Redundancy Point Optimization

In path planning algorithms, particularly when applying the A* algorithm to path search tasks, the generated path may contain redundant nodes. To improve path quality while ensuring path feasibility and obstacle avoidance, the algorithm prunes redundant nodes with the aim of replacing multiple line segments with a single line segment whenever possible. The algorithm specifically considers the “jump points” defined in this study, marking all nodes involved in the jumps or obstacles as essential points to ensure that these actions are not removed during optimization. The core concept of the algorithm is as follows:

Step 1: Create an empty new path sequence *P_new_*. The starting point *P*_1_ of the original path *P* is added as the first node to *P_new_*. Initialize index pointer *i* ← 1, which points to the index of the last confirmed and retained node in *P_new_* within the original path *P*.

Step 2: Start a loop iteration, where each iteration uses *P_i_* as the root node, *P_i_*_+1_ as the node to be checked, and *P_i_*_+2_ as the target node, forming a sliding detection window. The core of the algorithm is to determine whether there is an unobstructed straight-line path between the root node *P_i_* and the target node *P_i_*_+2_. The two principles are as follows: (1) Confirm that the node to be checked, *P_i_*_+1_, is not a critical node that must be retained and that the target node *P_i_*_+2_ exists. (2) If *P_i_*_+1_ is not a critical node, use the *Bresenham* line algorithm to check whether there is a collision with obstacles when moving from *P_i_* to *P_i_*_+2_.

Step 3: Decisions are made based on the detection results from Step 2: If there are no obstacles detected from *P_i_* to *P_i_*_+2_, then node *P_i_*_+1_ is a redundant point. In this case, delete *P_i_*_+1_, the target node *P_i_*_+2_ is stored as the next critical point in the *P_new_* sequence, and the index pointer *i* is updated to *i* + 2; if there are obstacles detected from *P_i_* to *P_i_*_+2_, or if *P_i_*_+1_ is a retained critical node, then *P*_*i*+1_ is a turning point. Next, add it to the *P_new_* sequence and update the index pointer *i* to *i* + 1.

Step 4: Repeat Steps 2 and 3 until the detection window index exceeds the range of the original path *P*. The path retains only the starting point, the end point, necessary turning points, and jump points.

**Figure 5:**
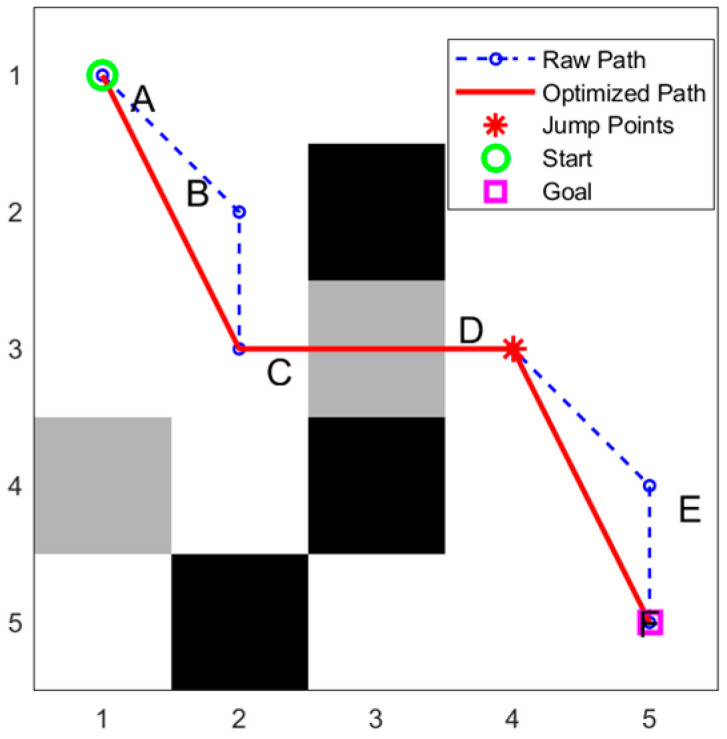
Comparison of the original path and the path after redundant point removal on a 5 × 5 simplified grid map.

It can be obviously seen from Figure 5 that A → B → C → D → E → F is the original obstacle crossing path, where C and D are jumping points. Considering three consecutive points A, B, and C on the path, if the line from A to C does not pass through any obstacle, then B is a redundant point. To ensure the continuity of the obstacle crossing action, obstacle crossing points C and D cannot be deleted. If there is no obstacle from D to F, the intermediate node E can be deleted, and finally, a simplified path is obtained: A → C → D → F.

### 2.7. Bezier Curve Smoothing

The A* path planning approach, built upon a graph search, was formed by multiple straight lines. The wheel-legged robot cannot change direction precisely during operation owing to hinge points. Bezier curves are required to optimize the path smoothness. The core equation is based on Bernstein basis functions:

(8) $$B(t)=\sum_{i=0}^{n}(\begin{array}{c} n\\ i \end{array}){\left(1-t\right)}^{n-i}t^{i}P_{i}\;(t\in [0,\;1]),$$

where *B*(*t*) is the curve trajectory function, *n* defines the curve’s degree, *P_i_* represents the control point, and *t* is a parameter. This study employed a second-order Bézier curve to enhance the path smoothness. Below is the form of the second-order Bezier curve:

(9) $$B(t)={\left(1-t\right)}^{2}P_{0}+2(1-t)P_{1}+t^{2}P_{2},\;t\in [0,\;1].$$

**Figure 6:**
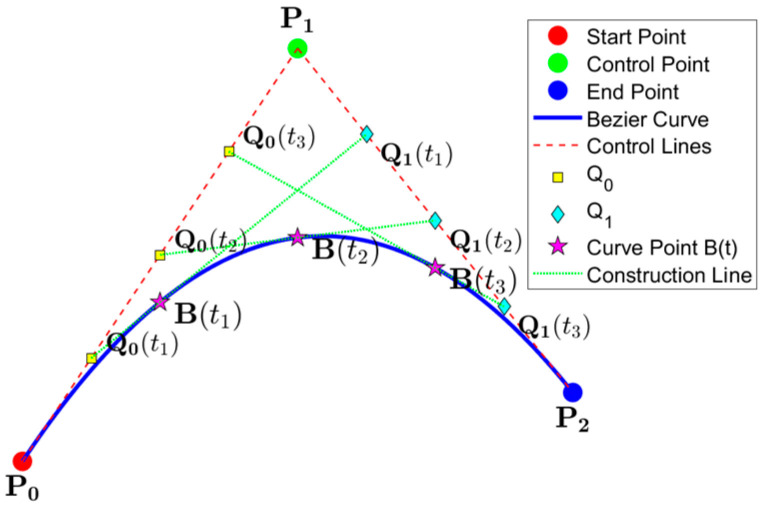
Diagram illustrating second-order Bézier curve optimization.

Figure 6 shows a diagram illustrating the second-order Bézier curve optimization, and the following are the exact steps:

Step 1: Extract nodes *P*_0_, *P*_1_, and *P*_2_ from the closed list to generate a polyline path.

Step 2: Select parameter *t* to calculate the middle point *Q*_0_(*t*), *Q*_1_(*t*) and move along the two line segments with *t*. As shown in Figure 6, take *t* = 0.25, 0.5 and 0.75 as follows:

(10) $$\left\{\begin{array}{l} Q_{0}(t)=(1-t)P_{0}+tP_{1}\\ Q_{1}(t)=(1-t)P_{1}+tP_{2} \end{array}\right.$$

Linearly interpolate between each pair of corresponding points *Q_0_*(*t*) and *Q_1_*(*t*) to determine the final point *B*(*t*) on the curve.

Step 3: Generate a Bezier curve defined by *P_i_, P_i+_*_1_, and *P_i_*_+2_ with redundant points removed. To ensure that the path is collision-free, the curve is discretized into a high-density sequence of intermediate points. The sampling parameter, *t*, is varied from 0 to 1 with a small step size (e.g., generating 30 points per curve segment) to create these points. Each discrete point on the curve is then mapped to its corresponding grid cell. The algorithm checks every cell for collisions with impassable obstacles (black cells).

Step 4: If the intermediate point, *P_i_*_+1_, is a designated jump point, it must be preserved to ensure the obstacle crossing maneuver is executed. In this case, the algorithm retains *P_i_*_+1_ and advances its window to start the next iteration from (*P_i_*_+1_*, P_i+_*_2_, and *P_i_*_+3_).

If *P_i_*_+1_ is not a jump point, generate a candidate second-order Bézier curve, *B*(*t*), using *P_i_, P_i+_*_1_, and *P_i_*_+2_ as the control points.

If the entire discretized curve is verified as collision-free, then the original sharp corner at *P_i_*_+1_ is replaced by the smoothed Bézier curve. The algorithm then advances its window in two steps to the next set of waypoints starting at *P_i_*_+2_. If any part of the curve is found to collide with an obstacle, the curve is discarded, waypoint *P_i_*_+1_ is retained, and the window advances by one step to the next iteration starting from *P_i_*_+2_.

Step 5: Repeat the process until all points in the path are processed. This ensures that the final smoothed path is continuous, avoids all obstacles, and preserves the required jump points.

While the introduction of Bézier curve smoothing and its associated collision-checking procedure introduces a minor computational overhead, its impact on real-time performance is negligible within the overall planning framework. This is because the smoothing is applied only to the final, significantly shortened path generated by the optimized A* search. However, these benefits are substantial in terms of the physical execution of the robot. The resulting smooth trajectory eliminates the need for abrupt stops and sharp turns at waypoints, which are kinematically inefficient and energetically expensive. By enabling the robot to maintain a more consistent velocity and reducing the frequency of acceleration/deceleration cycles, the smoothed path directly contributes to lower energy consumption and to reduced mechanical stress on the robot’s hardware, thereby enhancing the overall operational efficiency and longevity of the system.

## 3. Results

### 3.1. Experimental Environment Configuration

The proposed algorithm was validated using deterministic simulations. Similar to the traditional A* algorithm, it consistently produced the same path and performance metrics for any static environment with defined starting and goal points. Unlike stochastic algorithms, which require multiple runs for statistical significance, repeated trials on the same map yielded identical results. However, we conducted ten repeated trials. Thus, our validation focused on a comprehensive comparative analysis of different algorithm versions and a sensitivity analysis of the parameters under various conditions. This approach rigorously assesses the performance, robustness, and effectiveness of the proposed improvements without requiring statistical analysis of repeated runs.

This experiment was implemented in MATLAB 2022a under the hardware environment of a Windows 11 system, a 3.20 GHz AMD Ryzen 7 6800 H CPU, and 16 GB of RAM. A 40 × 40 grid map was constructed and static black insurmountable obstacles and gray surmountable obstacles were randomly generated.

### 3.2. Verification of the Effectiveness of Obstacle Surmounting Capability

**Figure 7:**
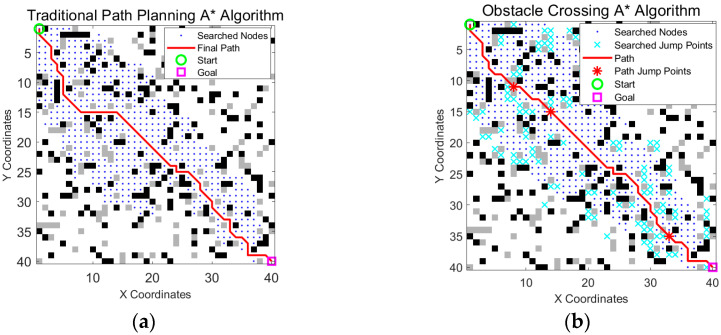
Comparison between the traditional A* algorithm and obstacle crossing A* algorithm. (**a**) The traditional A* algorithm based on obstacle avoidance; (**b**) obstacle crossing A* algorithm incorporating the unique jumping capability of wheel-legged robots. The green circle is the start point, the magenta square is the goal point, blue dots indicate searched nodes, cyan crosses represent all explored jump points, the solid red line is the optimized path, and the red stars on the path mark actual jump points.

To evaluate the performance advantage of the enhanced A* algorithm in the wheel-legged robot obstacle traversal task, this study carried out a comparative experiment using a 40 × 40 grid map. The environmental settings were as follows: the total obstacle density was 20% (black obstacles 12%, gray obstacles 8%), and the starting location (1, 1) and target point (40, 40) were fixed. The experiments were divided into two groups: the traditional A* algorithm and the obstacle crossing A* algorithm, as shown in Figure 7. As described in Section 2.4, the cost of horizontal/vertical jumping was assigned a constant value of 2, and the cost of diagonal blending was assigned a constant value of 2.8.

As shown in Figure 7 and Table 1, the traditional A* algorithm was compared with the obstacle crossing A* algorithm. Table 1 provides a comparison of the path length, search range, total number of jumps, and time consumption of the two algorithms in the above map. According to the simulation results, the traditional A* algorithm is forced to detour in areas with dense gray obstacles because it cannot utilize jumping actions. Owing to the obstacle crossing performance of the wheel-legged robot, adding an obstacle crossing to the A* algorithm can cross gray obstacles and thus greatly reduce the path length.

### 3.3. Multi-Scenario Adaptability Evaluation

**Figure 8:**
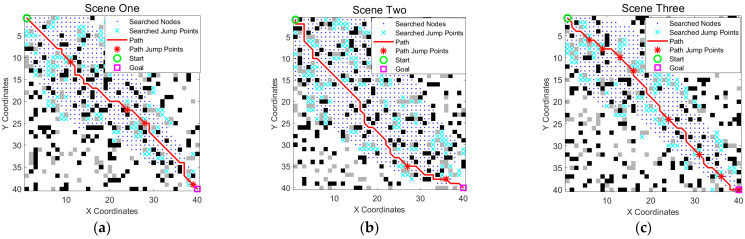
Effects of the obstacle crossing A* algorithm in different scenarios. (**a**–**c**) represent three different obstacle scenarios, respectively. The green circle is the start point, the magenta square is the goal point, blue dots indicate searched nodes, cyan crosses represent all explored jump points, the solid red line is the optimized path, and the red stars on the path mark actual jump points.

To assess the function of the obstacle crossing A* algorithm more accurately, three different scenarios were used to conduct the experiments, as shown in Figure 8 and Table 2.

As shown in Figure 8 and Table 2, the improved obstacle crossing A* algorithm showed good path planning capabilities in different scenarios. Although the path length increased slightly in some scenarios (such as 63.35 in Scenario 2), it performed stably in terms of path node optimization, search range compression, and jumping ability, effectively balancing the path quality and computational efficiency. This also demonstrates that the algorithm has good environmental adaptability.

Further analysis of the three paths shows that, even under different obstacle distributions, the paths generated by the algorithm have structural differences; however, they can effectively avoid obstacles by introducing a jumping mechanism to form a shorter and smoother path. This is attributed to the deterministic search framework and jump point guidance mechanism adopted by the algorithm, which can efficiently complete path search in static complex terrain, has good real-time stability, and can meet the needs of wheel-legged robots for fast path planning in complex environments. In particular, in Scenario 3, due to the presence of obstacles around the endpoint, which are gray and crossable, conventional obstacle avoidance would not allow reaching the destination. However, by incorporating the unique obstacle crossing capability of the wheel-legged robot, this feature can be utilized to reach the endpoint, thereby validating the effectiveness of the algorithm presented herein.

### 3.4. Comparison of the Obstacle Span Performance

As described in Section 2.3, we incorporated the unique obstacle crossing mechanism of wheel-legged robots into the traditional A* algorithm, enabling them to easily cross single-grid low-height obstacles. To verify this performance improvement, we conducted a comparative experiment by crossing two consecutive obstacles in the same scenario.

**Figure 9:**
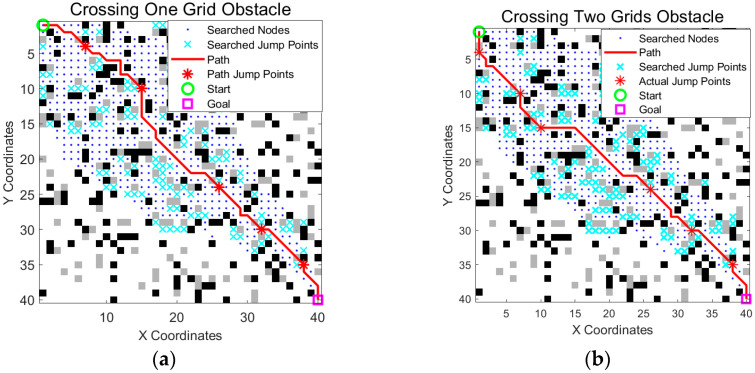
Comparison of the crossing one obstacle and crossing two obstacles scenarios. (**a**) Crossing a single obstacle cell in a horizontal, vertical, or diagonal direction; (**b**) crossing two consecutive obstacle cells in a horizontal, vertical, or diagonal direction. The green circle is the start point, the magenta square is the goal point, blue dots indicate searched nodes, cyan crosses represent all explored jump points, the solid red line is the optimized path, and the red stars on the path mark actual jump points.

The experimental results (Figure 9 and Table 3) show that while crossing two consecutive obstacles can slightly shorten the path length and reduce the number of path nodes such a maneuver involves greater kinematic complexity and risk. In practical applications, increasing the jump distance can negatively affect the landing stability of the robot and the overall success rate of the action. Therefore, despite the marginal path improvement shown in the simulation, we ultimately chose to retain the more reliable single-grid obstacle crossing solution to ensure higher motion stability and robustness in real-world scenarios.

### 3.5. Dynamic Heuristic Function Optimization

**Figure 10:**
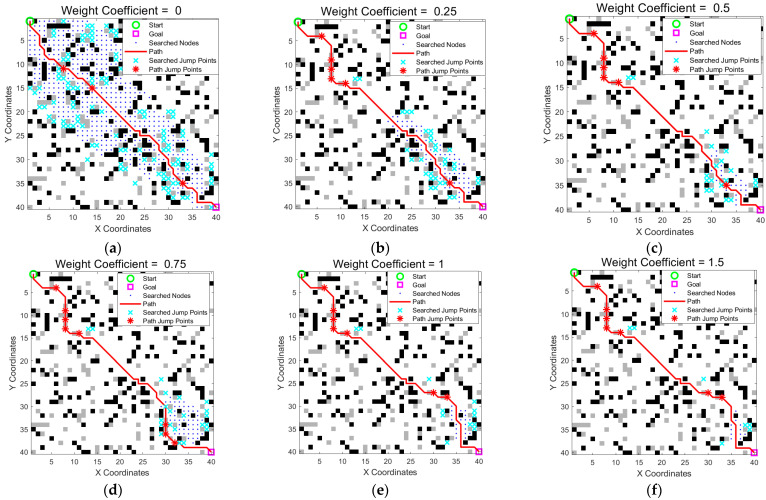
Path comparison under different weight coefficients. (**a**) With *α* = 0, the search covered a wide area. (**b**–**f**) As *α* increased, the search range was significantly reduced, resulting in a more focused and goal-directed path. The green circle is the start point, the magenta square is the goal point, blue dots indicate searched nodes, cyan crosses represent all explored jump points, the solid red line is the optimized path, and the red stars on the path mark actual jump points.

As discussed in Section 2.4, the dynamic adjustment coefficient *α* controls the balance between the search efficiency and path optimality. While the obstacle crossing mechanism improves the path directness, the search scope can remain large, as shown in Table 1. To address this issue, we introduced a dynamic heuristic weighting strategy. To determine an appropriate value for *α*, we conducted a sensitivity analysis by running the algorithm in the same scenario with the *α* set to various values: 0, 0.25, 0.5, 0.75, 1.0, and 1.5. The results are shown in Figure 10 and Table 4.

The data clearly demonstrate a trade-off controlled by *α*. As *α* increases, the heuristic becomes more aggressive, which significantly reduces the search scope (number of expanded nodes) and the planning time. For instance, increasing *α* from 0 to 0.5 reduces the search scope from 547 to 80 nodes—an 85.3% reduction. However, this gain in efficiency comes at the cost of a slightly longer suboptimal path (path length increases from 61.01 to 63.35, i.e., a 3.8% increase). When *α* is increased beyond 0.5, the gains in search scope reduction become marginal, whereas the path quality may degrade further. Therefore, we selected *α* = 0.5 as it offered an excellent compromise, drastically improving the search efficiency while maintaining a near-optimal path.

### 3.6. Verification of the Continuous Obstacle Crossing Constraint Mechanism

As shown in Section 3.5, after adding dynamic heuristic weights, the search range was significantly reduced, but continuous obstacle crossing occurred owing to an increase in the initial search speed. To verify the effectiveness of the continuous obstacle crossing constraint mechanism proposed in Section 2.5, the improved obstacle crossing A* algorithm with the constraint mechanism enabled was compared with the baseline algorithm without the mechanism enabled.

**Figure 11:**
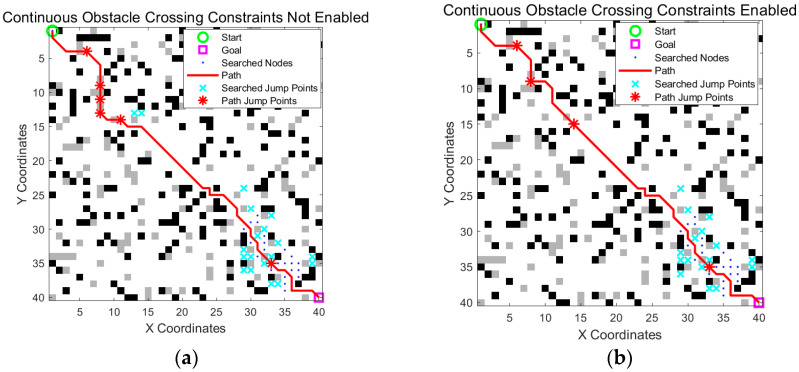
Verification of the continuous obstacle crossing constraint mechanism. (**a**) Without the constraint mechanism, the path exhibited multiple consecutive obstacle crossing actions, which may lead to instability; (**b**) with the constraint enabled, consecutive crossings were limited, resulting in more controlled and safer navigation. The green circle is the start point, the magenta square is the goal point, blue dots indicate searched nodes, cyan crosses represent all explored jump points, the solid red line is the optimized path, and the red stars on the path mark actual jump points.

From Figure 11 and Table 5, we can see that enabling the constraint did not significantly cause the path to detour. Instead, the path was optimized because of a more reasonable jump arrangement. When the path quality was preserved, the jump count was significantly minimized, the motion stability of the robot was improved, and the potential for dynamic instability caused by high-frequency continuous jumps was effectively avoided.

### 3.7. Comprehensive Performance Comparison Analysis

#### 3.7.1. Analysis of a 40 × 40 Grid

To address the requirements of wheel-legged robots for obstacle crossing and avoidance in complex terrains, this study proposes an improved A* algorithm that integrates a jump point search mechanism and a dynamic weighted heuristic strategy. The algorithm expands the traditional 8-neighborhood search to support multi-directional jump paths (horizontal, vertical, and diagonal) and combines this with refining the path by eliminating unnecessary points and applying Bézier curve smoothing to effectively optimize the performance and smoothness of the planned path.

**Figure 12:**
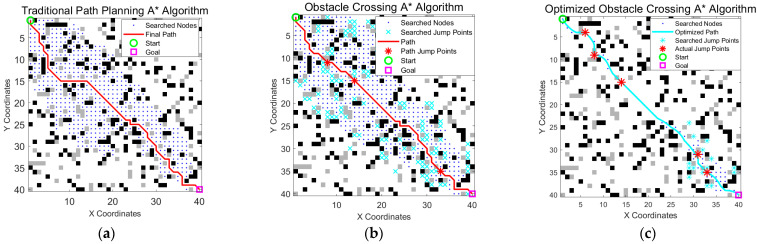
Algorithm path in a 40 × 40 map. (**a**) The traditional A* algorithm generated a path that avoided obstacles but tended to be longer and less smooth; (**b**) the obstacle crossing A* algorithm reduced path length by allowing jumps over gray obstacles; and (**c**) the optimized A* algorithm improved path continuity and smoothness, enabling more efficient robot traversal. The green circle is the start point, the magenta square is the goal point, blue dots indicate searched nodes, cyan crosses represent all explored jump points, the cyan line shows the optimized final path, and the red stars on the path mark actual jump points.

In the experiments, obstacles were categorized as insurmountable (black) and surmountable (gray), allowing the algorithm to achieve unified integration of avoidance and crossing strategies through differentiated processing. A comprehensive performance comparison showed that, in a 40 × 40 grid map, the fully optimized algorithm demonstrated significant improvements over the traditional A* algorithm. From Figure 12 and Table 6, the number of path nodes was reduced from 54 to 16 (a 70.4% reduction), the number of search nodes decreased from 542 to 78 (an 85.6% reduction), and the path length was also shortened. These results highlight the strong real-time performance and environmental adaptability of the algorithm.

#### 3.7.2. Scalability Analysis

**Figure 13:**
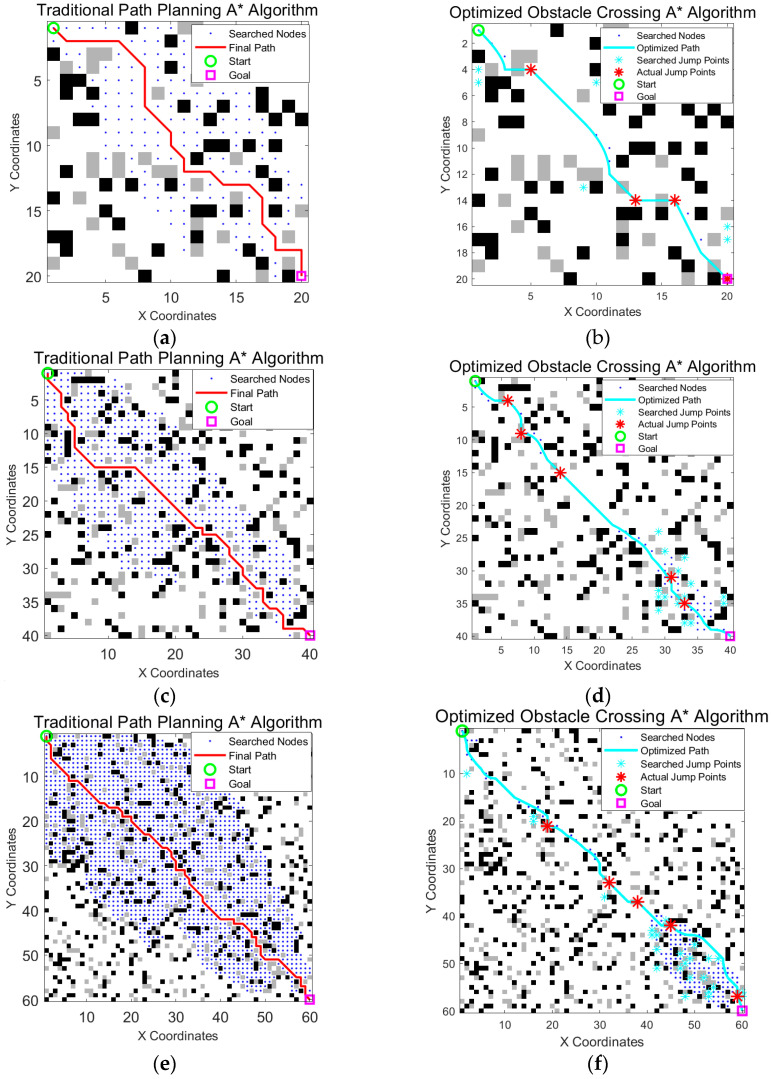
Comparison of the traditional A* algorithm and the obstacle crossing A* algorithm in this paper used on different map sizes: (**a**,**b**) are for 20 × 20 maps, (**c**,**d**) are for 40 × 40 maps, and (**e**,**f**) are for 60 × 60 maps. The green circle is the start point, the magenta square is the goal point, blue dots indicate searched nodes, cyan crosses represent all explored jump points, the cyan line shows the optimized final path, and the red stars on the path mark actual jump points.

To test the performance of the algorithm under different map sizes and obstacle densities, we maintained a constant obstacle density (20%) and ran the optimized algorithm and traditional A* algorithm on maps of various sizes, such as 20 × 20, 40 × 40, and 60 × 60, as shown in Figure 13 and Table 7.

The experimental results clearly demonstrate that the superiority of the optimized algorithm over the traditional A* algorithm became more pronounced as the map size increased. As the map expanded from 20 × 20 to 60 × 60, our proposed algorithm consistently maintained a significant advantage in reducing the number of search nodes and path nodes while also generating shorter and more efficient paths. This trend highlights the excellent scalability and robustness of our method, confirming its suitability for path planning tasks in larger and more complex environments.

## 4. Discussion

Aiming at the obstacle crossing and avoidance requirements of wheel-legged robots in complex terrains, this study proposes an improved A* algorithm that integrates a jump-point search mechanism and a dynamic weighted heuristic strategy. The algorithm expands the traditional 8-neighborhood to support multi-directional jump paths (horizontal, vertical, and diagonal) and combines redundant point removal with Bézier curve smoothing to effectively optimize the performance and smoothness of the planned path. In the experiments, obstacles were categorized as insurmountable (black) and surmountable (gray), allowing the algorithm to achieve unified integration of avoidance and crossing strategies through differentiated processing. The experimental results are compelling. In a 40 × 40 grid map, the improved algorithm reduces the number of path nodes by 73.7% (from 54 to 16) and shrinks the search range by 85% (from 542 to 78 nodes), with a planning time of only 0.0032 s, when compared to the traditional A* algorithm. Furthermore, a scalability analysis confirmed that the superiority of the algorithm becomes more pronounced as the map size increases, highlighting its excellent robustness and strong real-time performance for practical applications in larger environments.

This study validated the effectiveness of the proposed algorithm through simulation experiments in a 2D grid map environment. These results serve as a foundational validation of the algorithm’s core logic. The 2D grid map is a simplification and abstraction of the real world, where gray, crossable obstacles simulate barriers that can be traversed with a jump, such as rubble or low mounds, whereas black, non-crossable obstacles represent features that cannot be jumped, such as walls or high steps; however, despite this, we acknowledge the limitations of the current MATLAB-based 2D simulations. A key simplification in this study is the treatment of the robot as a point particle, which intentionally abstracts away complexities, such as robot dimensions, kinematics, dynamics, and precise foot placement, during jumps. This abstraction is a common practice in foundational path planning research to isolate and validate the core logic of the algorithm. The 2D grid map is a further simplification of the real world, where gray, crossable obstacles simulate barriers that can be traversed with a jump, such as rubble or low mounds, while black, non-crossable obstacles represent features that cannot be jumped, like walls or high steps. To fully substantiate the engineering applicability and motion stability of the algorithm, further validation on a physical robot platform or within a high-fidelity physics-based simulator, such as Gazebo, is essential. This constitutes the core of our future research.

Future research will focus on bridging the gap between current simulations and real-world applications. Furthermore, we recognize that the current study was confined to static environments. The next step is to extend the applicability of the framework to dynamic scenarios. This will involve integrating our high-level strategic planner with reactive, real-time collision avoidance algorithms, such as the Dynamic Window Approach (DWA), to create a hybrid planning system capable of navigating environments with moving obstacles. To overcome the limitations of this study, we developed a physical wheel-legged robot prototype for deploying the proposed algorithm. The next phase involved integrating a stereo camera to enable real-time 3D environmental perception, allowing for a transition from simplified 2D grid maps to more realistic 3D point cloud representations. Consequently, obstacles were defined by their actual physical dimensions, such as height and slope, rather than as a binary “crossable/non-crossable” state. The jump cost function was further refined by integrating the robot’s dynamic model to ensure that all planned maneuvers were both physically feasible and stable. This future work will significantly advance the practical applicability of the algorithm for complex missions, such as search and rescue or planetary exploration.

## 5. Conclusions

This study successfully developed and validated an improved A* algorithm tailored to the unique path planning challenges of wheel-legged robots in complex, static environments. By introducing an obstacle crossing mechanism with a 16-neighborhood search, a dynamic heuristic weighting strategy, and a continuous jumping constraint, the proposed algorithm fundamentally enhances the robot’s ability to intelligently decide between detouring around or jumping over obstacles. Further refinements, including refining the path by eliminating unnecessary points and applying Bézier curve smoothing, ensure that the final path is efficient.

The comprehensive simulation results demonstrate the significant superiority of our method over the traditional A* algorithm. In a 40 × 40 grid map, the optimized algorithm achieved a 73.7% reduction in path nodes and an 85% reduction in search nodes, all while generating a shorter and smoother trajectory. Scalability analysis confirmed that these performance gains become even more pronounced in larger environments, highlighting the robustness and efficiency of the algorithm. By equipping the path planner with a strategic obstacle negotiation capability, this study provides a robust foundation for enhancing the autonomous navigation and operational effectiveness of wheel-legged robots in challenging terrains.

## Figures and Tables

**Table 1:** Comparison between the traditional A* algorithm and obstacle crossing A* algorithm.

	Path Length	Path Nodes	Search Scope	Number of Jumps		Time Consumed (s)
Traditional A* Algorithm	63.355	54	542			0.0146
	
Obstacle Crossing A* Algorithm	61.012	47	547	3		0.0085

**Table 2:** Quantification of the performance indicators of the obstacle crossing A* algorithm in different scenarios.

Scenario	Path Length	Path Nodes	Search Scope	Number of Jumps
Scene 1	60.42	44	505	4
Scene 2	63.35	52	691	2
Scene 3	59.84	40	486	8

**Table 3:** Comparison of the crossing one-grid obstacle and crossing two-grid obstacle scenarios.

	Path Length	Path Nodes	Search Scope	Time Consumed
One grid	61.59	46	542	0.0134
Two grids	61.01	43	520	0.0194

**Table 4:** Comparison of the different weight coefficient parameters.

Weight Coefficient	Path Length	Path Nodes	Search Scope	Number of Jumps
0	61.01	47	547	3
0.25	63.35	48	137	6
0.5	63.35	48	80	6
0.75	64.52	48	110	8
1	65.11	50	74	7
1.5	65.11	50	65	7

**Table 5:** Quantification of the verification indicators of the continuous obstacle crossing constraint mechanism.

	Path Length	Path Nodes	Search Scope	Number of Jumps
Constraints not enabled	63.35	48	80	6
Constraints enabled	62.18	48	78	4

**Table 6:** Comparison of the simulation results obtained from a 40 × 40 map.

	Path Length	Path Node	Number of Jumps	Search Nodes
Traditional A* Algorithm	63.35	54	0	542
Obstacle Crossing A* Algorithm	61.01	48	3	547
Optimized Obstacle Crossing A* Algorithm	57.96	16	4	78

**Table 7:** Comparison of the quantitative metrics for the two algorithms used on different map sizes.

Map	Algorithm	Path Length	Path Nodes	Number of Jumps	Search Nodes
20 × 20	A* algorithm	32.72	30	0	184
	A* algorithm in this paper	29.04	8	4	23
40 × 40	A* algorithm	63.35	54	0	542
	A* algorithm in this paper	57.96	16	4	78
60 × 60	A* algorithm	96.91	83	0	1670
	A* algorithm in this paper	89.91	21	5	198

## Data Availability

No new data were created or analyzed in this study. Data sharing was not applicable in this study.
